# Mining multi-item drug adverse effect associations in spontaneous reporting systems

**DOI:** 10.1186/1471-2105-11-S9-S7

**Published:** 2010-10-28

**Authors:** Rave Harpaz, Herbert S Chase, Carol Friedman

**Affiliations:** 1 Department of Biomedical Informatics, Columbia University, 622 West 168th St., VC5, New York, NY 10032, USA

## Abstract

**Background:**

Multi-item adverse drug event (ADE) associations are associations relating multiple drugs to possibly multiple adverse events. The current standard in pharmacovigilance is bivariate association analysis, where each single drug-adverse effect combination is studied separately. The importance and difficulty in the detection of multi-item ADE associations was noted in several prominent pharmacovigilance studies. In this paper we examine the application of a well established data mining method known as association rule mining, which we tailored to the above problem, and demonstrate its value. The method was applied to the FDAs spontaneous adverse event reporting system (AERS) with minimal restrictions and expectations on its output, an experiment that has not been previously done on the scale and generality proposed in this work.

**Results:**

Based on a set of 162,744 reports of suspected ADEs reported to AERS and published in the year 2008, our method identified 1167 multi-item ADE associations. A taxonomy that characterizes the associations was developed based on a representative sample. A significant number (67% of the total) of potential multi-item ADE associations identified were characterized and clinically validated by a domain expert as previously recognized ADE associations. Several potentially novel ADEs were also identified. A smaller proportion (4%) of associations were characterized and validated as known drug-drug interactions.

**Conclusions:**

Our findings demonstrate that multi-item ADEs are present and can be extracted from the FDA’s adverse effect reporting system using our methodology, suggesting that our method is a valid approach for the initial identification of multi-item ADEs. The study also revealed several limitations and challenges that can be attributed to both the method and quality of data.

## Background

The main objective of pharmacovigilance is the early detection of novel adverse drug events (ADEs) with minimal patient exposure. The impact of ADEs results in significant social costs estimated in several billion dollars annually, and inflicts unnecessary, often fatal, harm to patients [1,2]. Hence, their identification is paramount to health care.

Spontaneous reporting systems (SRS) are database resources encompassing reports of suspected post-marketed ADEs, and are currently the mainstay in pharmacovigilance. Among the major SRSs are: the United States Food and Drug Administration’s (FDA) Adverse Event Reporting System (AERS) [3], and the World Health Organization (WHO) Programme for International Drug Monitoring [4].

The FDA receives voluntary reports of suspected adverse drug events directly from health care professionals and consumers, as well as mandatory reports from manufacturers, which after a manual review are entered into the AERS Database. Each report contains patient demographic information, drug information for as many medications as were reported for the event, including suspected drugs and concomitant drugs, coded adverse events using the MedDRA terminology [5] (a terminology developed for ADE applications), patient outcomes, drug therapy dates, MedDRA coded indications for the reported drugs, and report sources. The AERS Database, which is available online, contains over four million reports of adverse events and reflects data from 1969 to the present.

Traditional methods of ADE detection in SRS databases relied on manual case reviews by clinical/pharmacological experts. However, due to the increasing size and complexity of SRS databases, and limitations in human resources, recently, more efficient methods have been proposed consisting of automated, and quantitative approaches that are commonly referred to as data mining algorithms (DMAs) or signal detection algorithms. DMAs are generally designed to identify statistically strong associations between drugs and adverse effects (AEs). These associations, also referred to as signals, are not necessarily true ADEs but rather hypotheses that warrant further investigation to qualify them as credible ADEs. They allow evaluators to peruse the large volume of reports and focus their attention on potentially important safety issues.

In recent years a wide range of DMAs have been developed to screen potential ADEs [6-8]. To estimate incidence rates, DMAs use the population of reports in the SRS as a proxy for the true population. The majority of DMAs rely on the use of disproportionality measures, such as the relative reporting ratio (RR), which attempt to quantify the degree of “unexpectedness” of a drug-AE association [9]. Typically, a pre-defined disproportionality threshold will be used to screen potential ADEs for further review. Both the FDA and WHO use an adjusted version of RR as a basis for monitoring safety signals in their SRS [10,11].

Typical SRS databases contain tens of thousands of drugs and adverse effects (AEs). Enumerating all possible combinations of ADEs for statistical analysis, although feasible, is a daunting task and rarely done. As a result, most published work focuses on subsets of the data, e.g., specific drugs or demographic groups, avoiding data base wide studies. Additionally, most current DMAs are designed to identify only binary (bivariate) associations, i.e., pairs including only one drug and one AE, such as

Vioxx→ heart attack,

excluding from analysis the possibility of multi-item associations, e.g. an association between two drugs and one or two AEs, such as

Aspirin + Warfarin → Bleeding.

Multi-item ADE associations are rarely reported but are important because they could indicate possible drug-drug interactions. The limitations of bivariate analysis, as well as the importance of and difficulty of multi-item ADE detection, also referred to as higher-order ADE associations, was emphasized in [6,12] noting that SRS databases provide an opportunity to uncover them as they contain populations that are not well represented in clinical trials. Studies that did consider multi-item ADE associations, such as drug-drug interactions, did so only after a careful selection of a small subset of drugs [13,14].

Association rule mining is a well established method for discovering interesting relationships between variables hidden in large databases. An association rule is an implication expression of the form *A→B*, where *A* and *B* are disjoint itemsets. In the case of ADE detection, *A* denotes a set of drugs and B a set of AEs, e.g. *A= Aspirin*, *Warfarin*, *B= Bleeding*. The strength of an association rule is determined by its *support* and *confidence*. The support of an itemset *S(A)* is the number of records containing *A*. The support of an association rule *S(A→B)* is equal to *S(A*∪*B)*, i.e., support determines how often a rule, which in this case is the combination of drugs and AEs, is observed in the data. Low support may indicate that a rule has simply occurred by chance, and thus support is often one of the parameters used to screen uninteresting rules. The confidence of a rule *C(A→B)* is equal to *S(A*∪*B)/S(A)*, that is, confidence determines how often items in *B* appear in records that contain A. Confidence provides an estimate of Pr*(B|A)* the conditional probability of *B* given *A*, and therefore is used to measure the reliability or interestingness of the rule. It should be emphasized that the inference made by an association rule does not imply causality. Nevertheless, association rule mining provides a natural setting for ADE detection and analysis, and in recent years, has been adopted to various problems in the area of biomedical surveillance. Brossette et al. and Ma et al. [15,16] applied association rule mining to hospital infection control. Chen et al. [17] applied association rules mining to a linked dataset comprised of a pharmaceutical prescribing dataset and a hospital admissions dataset in order to identify groups of patients who are more likely to have an adverse effect to ACE inhibitors. Rouane et al. applied [18] association rules mining to identify ADEs related to anti-HIV drugs.

Two key issues need to be addressed when applying association rule mining. First, the search space of interesting multi-item associations is extremely large, and discovering these associations in large databases is computationally expensive, often intractable. For example, assuming 10,000 unique drugs and AEs are under consideration (a common scenario), then the number of possible multi-item associations made of 2 drugs and 3 AEs that need to be examined is approximately 10,000^5^ =10^20^ , and for each, incidence rates and other association statistics need to be computed. Second, some of the associations discovered may be spurious (happening by chance), or due to confounding factors. Both of these issues have been addressed to a certain extent in this work.

The objective of this paper is twofold. First, to demonstrate the feasibility and value of association rule mining to identify interesting multi-item ADEs. Second, to demonstrate that multi-item ADEs exist in the AERS database and can be discovered by our method. Mining the AERS database for multi-item associations is to the best of our knowledge a study that has not yet been done for the scale and generality proposed in this paper. Unlike other studies, our approach is general and is not restricted to a particular group of drugs or set of specific conditions, while examining tens of thousands of reports submitted over several years. The exploration of such a large space of possible multi-item associations was made possible partly due to an optimized and tailored implementation of the Apriori [19] algorithm. We propose the use of association rule mining as an important and promising first step in a multi-step process where subsequent steps include rigorous statistical analysis and clinical/pharmacological expert judgment.

## Methods

### Data sources

For the purpose of this study we selected a large sample of reports published in the year 2008, representing a sample from the latest complete set of yearly AERS reports available. Selecting from the latest available set of reports offers the opportunity to study novel multi-item ADEs. In addition we selected reports which were categorized as having a “serious” patient outcome (death, life-threatening, hospitalization, disability, etc), and reports which contained more than one drug. The reason for the latter being that we wanted to explore multi-item associations corresponding to possible drug-drug interactions.

### Mining process

**Figure 1:**
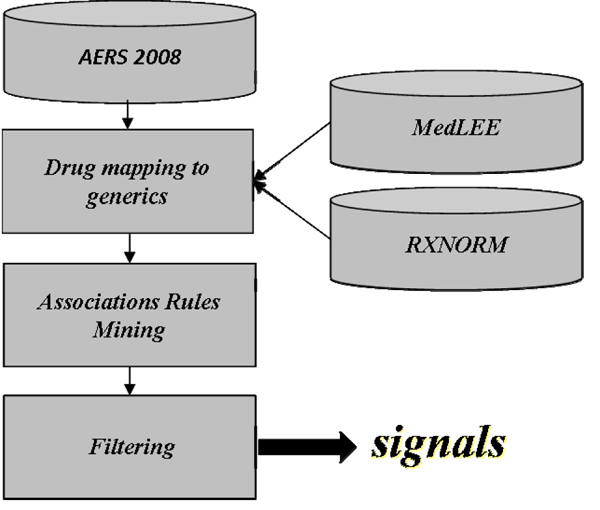
Overall mining process

The overall mining process consisted of three steps depicted in Figure 1. (1) drug names were mapped to their corresponding generic names to reduce drug naming redundancy and strengthen the signals, as well as reduce algorithmic complexity, (2) a set of candidate multi-item ADE associations were generated using an optimized and tailored implementation of the Apriori algorithm, (3) the set of candidate multi-item ADE associations generated in the second step were filtered to remove spurious associations, whereupon the final set of potential multi-item ADEs were obtained.

Our method was implemented in Python and required approximately 4 hours to complete a full run on a standard desktop with an Intel Core 2 Duo processor running at 3.0GHz.

### Drug name mapping

One of the main challenges presented to DMAs is the granularity and large variation in the terminology used to describe drugs and adverse effects in AERS, which in turn may dilute ADE signals across multiple similar drugs or events. Unlike the suspected ADEs and the indications which are coded using MedDRA, the drugs are entered as free text and include a variety of different formats as well as typographical errors, which must first be mapped into a standardized form. For example, in AERS 2008, there were several dozen different variations for the drug Avandia, some of which were: AVANDIA, AVANDIA (ROSIGLITAZONE MELEATE), AVANDIA /SCH/ (PIOGLITAZONE HYDROCHLORIDE), AVANDIA /UNK/ (ROSIGLITAZONE MALEATE), AVANDIA (CON.), AVANDIA /01445801/ , AVANDIA /SCH/ (PIOGLITAZONE HYDROCHLORIDE) , AVANDIA /SCH/ (ROSIGLITAZONE MALEATE) , AVANDIA (2 MILLIGRAM TABLETS), and ROSIGLITAZONE MALEATE (AVANDIA).

Each drug obtained from a report was first assigned a UMLS drug code using MedLEE[20], an existing NLP system. If the drug name included a dose or route, e.g. “Avandia (2 milligram) tablets”, the more general UMLS code consisting of only the drug name was chosen over the more specific code, e.g., C0875967, corresponding to “Avandia”. If the drug name could not be mapped, it was left as is. Finally, UMLS codes were mapped to generics using RXNORM[21]. Hence, C0875967 corresponding to the brand name Avandia, would be mapped to C0289313 corresponding to the generic name for Rosiglitazone.

### Associations rules mining

The space of possible multi-item associations in a large database, such as the AERS database, is generally so large that the number of possible rules to search over is computationally intractable. Therefore, it was necessary to use an efficient algorithm, and also to employ additional criteria specific to the application, in order to reduce the search space. These additional criteria restrict the search space to rules that not only have high support and confidence but that are of high priority to the application, such as rules containing a certain number or set of items. In this work we used the general Apriori algorithm, which was optimized and tailored to this specific application.

The Apriori algorithm is a method designed to efficiently identify association rules in large databases. The Apriori algorithm prunes the search space of associations based on the basic downward closure property of frequency. In our context this means that if a certain combination of drugs and AEs is infrequent, then any larger combination that builds upon the smaller infrequent one, will also be infrequent, and thus need not be considered. For example, if the combination of drugs aspirin and metformin is in frequent, then any association rule that builds upon it, such as aspirin + metformin*→*headache, will also be infrequent and therefore does not need to be considered.

The general Apriori algorithm works in two steps. The first step searches for item sets that have more than a given minimum support, while in the second step, rules are generated by selecting “confident” item sets (based on a threshold) from those found in the first step. It is the first step that presents the bottleneck and greater challenge, as it is in this step that all possible rules are enumerated. In order to deal with the very large set of reports from AERS and make the problem tractable we made the following two changes and enhancements to the basic Apriori configuration.

1. Imposing the constraint that only item sets that had a set of drugs in the antecedent and a set of AEs in the consequent were considered as potential association rules in the first step. Otherwise, many of the associations generated by the algorithm would contain either only drugs or only AEs, which is not aligned with the definition of an ADE association. By imposing this constraint the search space for possible multi-item ADE associations and candidate rules that needed to be considered was significantly reduced.

2. Instead of scanning the entire database in order to compute the support of each candidate association rule, hashing (indexing) based on drugs/AEs was implemented to reduce the number or reports that need to be examined for each rule.

The scale of computational gain achieved by these optimizations was several thousand folds, and without them the method was intractable.

### Filtering

Similar to other studies [15,16,18], we have found that the standard criterion of confidence is not indicative of the set of interesting rules. The inappropriateness of confidence for this case stems from the fact that frequent AEs, such as nausea, are likely to generate large confidence values regardless of the drugs associated with it, and infrequent AEs are likely to produce small confidence despite being strongly associated with certain drugs. Moreover, unlike typical applications of association rule mining the type of associations sought in ADE mining are by definition rare, especially multi-item associations, which would manifest themselves as rules having relatively low support and confidence. On the other hand, an ADE association with high confidence would most likely already be known, e.g., discovered during the clinical trial process. We therefore chose to use the relative reporting ratio (RR) as a proxy for rule interestingness and association strength instead of confidence.

RR is defined as the ratio between a rule’s observed frequency to a baseline expected frequency under the assumption of independence, the latter servings as a control. In terms of our existing definitions of support and confidence, RR can formally be defined as,

where *N* is the total number of reports in the data. In this setting *S(A*∪*B)* (support) is viewed as the observed frequency of a rule, and *S(A)S(B)/N* as its expected frequency under the assumption of independence. It is also easy to see that in this setting RR can be viewed as confidence normalized by the support of a rule’s consequent (B), correcting the shortcoming of confidence discussed above. In the context of ADE detection, high values of RR indicate that the occurrence of a set of adverse effects in conjunction with a certain set of drugs is larger than occurrence of the adverse effects in the general population of drugs. Under a probabilistic interpretation, RR is an estimate of

Therefore, RR can also be viewed as the amount of deviation of the joint probability of the drugs and AEs from statistical independence. Large values indicate that the occurrence of the drugs-AEs combination has unlikely occurred by chance and that a plausible reason is behind the association. Roughly, RR=1 indicates that the drugs and AEs are statistically independent, RR>1 that the drugs and AEs are positively correlated, and RR<1 that the drugs and AEs are negatively correlated. Although having its limitations, as do other measures, we found RR to be a more informative criterion than confidence for screening interesting ADEs. Additionally, we found that an adjustment that accounts for low variance, such as the Gamma Poission Shrinker [22] used by the FDA, would not be necessary in this case due to a high enough support that we selected. For large enough support the adjusted and unadjusted RR are almost equal [22,23].

The support and RR thresholds used in this work to screen ADE association rules were set to 50 and 2 respectively. In the absence of a gold standard (the set of all true multi-item associations is unknown) which would have enabled us to calibrate or determine the most appropriate thresholds in a quantitative manner, we resorted to a data driven and heuristic approach guided by domain expert knowledge. The support threshold was set to a high enough value to highlight the more frequent patterns and at the same time to accommodate for the size of the database. The threshold (50) was found to be a balancing point between the number of associations generated, their size, and variation in content (drugs and AEs appearing in an association). Setting the threshold to a lower value resulted in a much larger set of associations at a more granular level (smaller), with a higher risk of containing spurious ones. A larger value resulted in less variation in content.

Although there is no consensus as to the most appropriate RR threshold [8], the RR threshold used in this work was set to the value of 2 based suggestions noted in similar studies [6,10,24]

### Evaluation

Following common practice in pharmacovigilance[6,8] the clinical validity of the multi-item ADE associations identified by the proposed method were reviewed by a clinical subject matter expert. The findings and conclusions were based on a random evaluation sample of 100 multi-item ADE associations. Known ADEs were validated by the expert using Micromedex [25], which is considered a reliable reference for medications and their associated ADEs.

## Results

### Data statistics

The full set of AERS reports published in 2008 contains 441,009 reports. Our overall data collection process resulted in a sample of 162,744 individual reports used for analysis, containing 24,641 unique drug names, and 8,025 MedDRA coded unique AEs. The step which mapped drug mentions to generics reduced the set of drug names from 24,641 to 7,094 unique UMLS coded drugs. Each report contained on average 3.3 different drugs associated with the report and an average of 3.4 different AEs, yielding an average number of 6.7 items per item set. The median number of different drugs and different AEs per report was 2 for each. It was found that 60% of the reports contained more than one drug, 70% of the reports contained more than one AE, and 84% of the reports contained at least 3 items (drugs or AEs) providing one of the main motivations for this study. Additionally, we found that 27% of the drugs were reported as the primary suspect for the AE, 15% as secondary suspect, 58% as concomitant, and that all reports in our sample were classified as having a “serious” patient outcome.

### Multi-item ADE Associations

Using the proposed thresholds our method produced a set of 2603 association rules. Of those, 1167 were multi-item associations containing item sets of size 3 or more, i.e., at least 2 drugs or 2 AEs.

Based on the evaluation sample of 100 associations, we developed a taxonomy that characterizes the set of multi-item ADE associations (association rules) that were identified. The taxonomy along with estimated proportions of each category in the taxonomy is presented in Table 1, whereas Table 2 provides representative examples of potential multi-item ADEs classified according to the taxonomy, together with the support and RR value for each.

**Table 1:** Taxonomy of multi-item ADE associations

Drugs		
1a	Drug-drug interactions found that are known	4%
1b	Drug-drug combinations known to be given together or treat same indication	78%
1c	Drug-drug combinations that seem to be due to confounding	9%
1d	Drug-drug interactions that are unknown	9%

**Associations**		

2a	Associations (drug[s]-event) that are known	67%
2b	Associations (drug[s]-event) that are unknown	33%

**Table 2:** Classified Sample of multi-item ADE associations found in AERS

	Taxonomy	Multi-item ADE Association	Support	RR
A	1a-2a	Metformin, Metoprolol -> NAUSEA	50	7.4
B	1b-2a	Cyclophosphamide, Prednisone, Vincristine -> FEBRILE NEUTROPENIA	78	45
C	1c-2a	Cyclophosphamide, Doxorubicin, Prednisone, rituximab -> FEBRILE NEUTROPENIA	63	59
D	1b-2b	Atorvastatin, Lisinopril -> DYSPNOEA	55	3.5
E	1a-2b	Omeprazole, Simvastatin -> DYSPNOEA	58	12
F	1d-2b	Varenicline, Darvocet -> ABNORMAL DREAMS, FATIGUE, INSOMNIA, MEMORY IMPAIRMENT,NAUSEA	52	2668

## Discussion

The taxonomy and examples provided in Table 2 illustrate several interesting patterns of drugs and adverse effect combinations that can be found in AERS.

The associations in examples A, B and C describe drug-drug interactions that are known, drugs that are frequently prescribed together, or associations that seem to be due to confounding respectively. For example, in association A, *Metformin* and *Metoprolol* are known drug-drug interactions, and because each is associated with nausea, it can be predicted that nausea is more common in patients taking both drugs. The three drugs identified in association B are used together to treat certain types of cancer and the ADE, *febrile neutropenia,* is a known complication of the treatment. *Prednisone* is given with the other drugs in association C to treat various medical conditions, such as to induce immunosuppression, but does not cause the reported outcome. Based on historical documentation, this suggests our method has the potential to uncover important ADEs that have not yet been recognized. Associations D and E describe drugs known to be given together or drug-drug interactions that are known (category 1a) with events that are clinically unknown (category 2b). Both drugs in association D are commonly prescribed together in patients with various medical conditions (such as *diabetes*), but the reported event *dyspnoea* is not expected. If *dyspnoea* was a typical manifestation of a disease (congestive heart failure due to diabetes), then it is likely that the report would not have been submitted to AERS. Association E reports the same ADE but with a different antecedent. There are preliminary reports that drugs of the first class (*omeprazole*) interact with metabolism and thus augment the action of drugs of the second class (*simvastatin*). The observed outcome, *dyspnea*, is not known to be due to either of these two drugs. If dyspnea were due to *congestive heart failure*, for which *Simvastatin* might be prescribed, it would be expected and not submitted to the AERS database as an adverse drug event.

After sorting the associations by their RR we came across a case that is reflective of data quality issues associated with the AERS database. Association F received the highest RR value among all associations found. The extremely high value (2669) assigned to it raised our suspicion, and its corresponding 52 reports (instances) were pulled from the AERS database for further inspection. Each of the patients was on 18 or more drugs. Yet, despite the very many combinations that would result from a random combination of these drugs, the varenicline-darvocet combination was much more commonly associated than any of the other combinations. Out of 62 reports containing both drugs, 52 also contained the AEs listed. Furthermore, only a small subset of 12 AEs were associated with these drugs. Six of them are related to mental functioning. This suggests that the method may have identified a potential drug-drug interaction which augments the effect of either on the brain. This has face validity: both of these drugs influence the central nervous system so that it is feasible that some of the AEs are related to the combination. The only caveat is that it appears that all patients are nearly identical; all have same diseases, all were taking nearly all of same drugs and reported nearly all of same AEs. This suggests duplicity in reporting of the event/s, a well known issue with AERS [9,26]. A plausible explanation for this duplicity is that different reporters (health care professionals, consumers, and manufacturers) reported the same patient, possibly from different arms of a clinical trail, or (and) that follow-up reports for the patient were not properly linked to the patient’s earlier reports. In any case, such duplicity may severely bias the statistics and lead to erroneous conclusions.

Our findings show that the majority of associations (78%) were composed of drugs that are usually given together, such as antibiotics, or drugs that treat the same disease, e.g. cancer (taxonomy 1b). The findings also show that the majority of associations (67%) were recognized (known) multi-item ADE associations, where each of the drugs is known to cause each of the reported adverse effect (taxonomy 2a). This finding demonstrates that multi-item ADE associations exist in AERS, and that our method was able to reveal them, in turn suggesting that our method is a valid approach for the initial identification of multi-item ADEs. We note that a substantial proportion of associations included ADEs that have not yet been recognized (taxonomy 2b). However, this does not necessarily indicate that these associations are incorrect, but that currently support for them is scarce and that further investigation is required.

A much smaller proportion of associations (4%) included drugs that are known to interact (taxonomy 1a). This may or may not be indicative of the true number of drug-drug interactions contained in AERS. The exact number is unknown, but there are studies[27] that suggest a larger number (6%-30%). Regardless, drug-drug interactions are a special case of multi-item ADEs, and our methodology was not designed for this specific case but for the more general case. Nonetheless, we believe that this methodology can be used as a basis for a method designed to identify drug-drug interactions, and we plan to investigate its potential in future work.

The experiment also revealed several limitations and challenges that can be attributed to both the method and quality of data. Based on statistical criteria, the method was able to expose multi-item potential ADEs that would otherwise go unforeseen by conventional methods, achieving its preliminary goal. However, without a rather tedious manual review of an expert none of the associations found could be validated or interpreted in the right context. Ideally, a DMA should be able reduce the amount of manual expert intervention by perhaps being able to classify the associations detected based on some taxonomy, such the one developed in this study. This however is still an open problem and beyond the scope of this study. In future research we intend to develop a DMA based on association rule mining supplemented by additional statistical methods that would reduce the amount of intervention and produce associations of higher quality. One aspect of this problem we are currently exploring is the detection of confounded associations.

There is no gold standard regarding which statistical measures are to be used in order to facilitate accurate ADE screening, especially multi-item ADEs, where little research has been reported. This problem is exacerbated in SRS databases due to unknown incidence rates in the general population. We believe it is likely that interesting associations went undetected by our method due the specific statistical measures we used. In future research we plan to explore the applicability, strengths and limitations of various measures to the application of multi-item ADE screening.

There are several algorithmic improvements that would enable our method to cope with a larger volume of associations. For example, lowering the support threshold would have produced a larger and more granular amount of associations, some of which would have likely been classified as interesting potential ADEs. At its current state this action would be prohibitive.

Due to the mostly voluntary nature of reporting in SRS databases DMAs are susceptible to biases inherent in the data [6,12]. Common phenomena include under reporting, and over reporting of AEs, and reporting duplicity as illustrated by association F in Table 1. There were other reports where over 50 AEs or drugs were included in one report. All these may generate spurious ADE association, and without taking into account data quality issues the results are questionable.

## Conclusions

In a comparative study of DMAs applied to pharmacovigilance [6] the authors state: ”If DMAs have value, it is because they achieve one or more of the following … detection of AEs that would otherwise have gone undetected (this is especially pertinent to higher order associations because they are difficult to be captured …)”. The current standard in pharmacovigilance is bivariate association analysis, where each drug-AE combination is studied separately. In this paper we propose a fundamentally different approach taking into account multi-variate associations. We examined the feasibility of a well known data mining method that we adopted and tailored to the problem of multi-item ADE detection in SRS. We demonstrated its incremental value as part of a larger scheme of operation, which would include additional statistical methods supplemented with domain expert knowledge. The method was applied in a database-wide-manner to FDAs AERS with minimal restrictions and no prior expectations on its output, an experiment which to the best of our knowledge has not been done previously. The findings demonstrate that multi-item ADEs are present and could be extracted from AERS using our methodology. There are several challenges concerning our approach that should be addressed. In future research we plan to address some of these issues such as: the statistical measures that should be used to qualify ADEs, algorithmic improvements, a more automated and streamlined detection process, and incorporating data quality into analysis.

## Authors' contributions

RH designed the study, collected data, implemented the algorithm, carried out the experiment, performed statistical analysis, and drafted the manuscript. HC participated in the design of the study, provided clinical domain expertise, validated results, and helped draft the manuscript. CF conceived of the study, participated in the design and coordination of the study, and helped draft the manuscript. All authors read and approved the final manuscript.

## Competing interests

The authors declare that they have no competing interests.
